# Sensory Evaluation of Foods with Added Micronutrient Powder (MNP) “*Taburia*” to Assess Acceptability among Children Aged 6–24 Months and Their Caregivers in Indonesia

**DOI:** 10.3390/nu9090979

**Published:** 2017-09-06

**Authors:** Aang Sutrisna, Marieke Vossenaar, Doddy Izwardy, Alison Tumilowicz

**Affiliations:** 1 Global Alliance for Improved Nutrition (GAIN), Menara Palma Lantai 5 Unit 502-B, Jln. HR. Rasuna Said Blok S-2 Kav. 6, Jakarta 12950, Indonesia; asutrisna@gainhealth.org; 2 Global Alliance for Improved Nutrition (GAIN), 7, Rue de Varembe, 1202 Geneva, Switzerland; mvossenaar@gainhealth.org; 3 Direktorat Gizi Masyarakat, Jln. HR. Rasuna Said Blok X-5 Kav. 4-9, Jakarta 12950, Indonesia; izwardydoddy@gmail.com

**Keywords:** micronutrient powders, sensory evaluation, infants and young children, Indonesia

## Abstract

Although it is generally accepted that the addition of micronutrient powders (MNPs) to foods causes no or negligible changes to organoleptic properties, there are anecdotal reports of low acceptability of the MNP (locally known as “*Taburia*”) distributed in Indonesia. We hypothesized that the organoleptic properties of *Taburia* do not reduce the acceptability of foods if used as recommended. Acceptability of *Taburia* and a comparison MNP (MixMe™) were evaluated among 232 children aged 6–24 months and their caregivers. Both caregivers’ perceptions of child acceptance, and their own assessments of organoleptic qualities when added to rice porridge or meals commonly consumed by young children, were assessed. Changes to the organoleptic properties of foods mixed with *Taburia* and comparison MNP were reported by caregivers, even when following preparation instructions. *Taburia* was found to enhance texture, sweetness, saltiness, and umami taste, but was also perceived as slightly bitter. Ratings for overall appearance and taste did not differ between rice porridge, plain or with *Taburia*, but the overall taste of *Taburia* was preferred over comparison MNP (*p* = 0.012). Meals consumed by children were preferred without the addition of MNP (*p* < 0.001). We demonstrate that the addition of *Taburia* to foods, commonly consumed by Indonesian infants and young children, affects organoleptic properties of the foods, even when prepared according to recommendations. However, these changes are unlikely to be the cause of reported adherence problems in Indonesia. This needs to be taken into consideration for product development and communication strategies promoting adherence.

## 1. Introduction

Food insecurity, malnutrition and anemia are persistent challenges in Indonesia, particularly in the eastern provinces [1,2]. The 2013 Indonesian national survey (Riskesdas) reports that over one-third (37%) of children under five years are stunted, one-fifth (20%) are underweight and almost one-third (28%) suffer from anemia [2]. Although not documented in peer-reviewed publications from Indonesia, iron deficiency is likely to be an important cause of anemia among Indonesian children [3,4,5,6,7,8].

Point-of-use fortification of complementary foods with iron-containing micronutrient powders (MNPs) is recommended by the World Health Organization (WHO) as a strategy to improve iron status and reduce anemia in infants and young children aged 6–23 months in populations where anemia is a public health problem [9]. MNPs were originally developed as an alternative to supplementation with iron drops, syrup or tablets in the prevention and treatment of iron deficiency anemia [10]. The impact of iron supplementation in these forms was limited by poor adherence due to gastrointestinal side effects, unpleasant and strong metallic taste, staining of teeth, and difficulties with dosing and swallowing [11,12]. MNP, available in single-dose sachets of dry powder containing lipid-encapsulated iron and other micronutrients, overcome most of these acceptability issues, when prepared as instructed [10,13].

In Indonesia, the National Institute for Health Research and Development (NIHRD) of the Ministry of Health (MOH) developed a local MNP named “*Taburia*”, currently (2017) being distributed across 3 of 34 provinces, and 64 of 514 districts. *Taburia* contains 14 micronutrients, including iron as ferrous fumarate (10 mg), and its formulation is regulated by technical specifications of the MOH (amended in 2013) to fulfill the daily micronutrient needs of young children, i.e., at least 100% of the WHO/Food and Agriculture Organization of the United Nations (FAO) recommended nutrient intakes (RNIs) [14].

It is widely accepted that MNP causes no or negligible changes to the organoleptic properties of foods to which they are added [9,13,15,16]. Although MNPs generally have high acceptability in field settings [17,18], there are documented reports of changes to sensory properties of foods with MNP. Studies describing the acceptability of the sensory attributes of MNP in community settings typically rely on the caregiver’s perceptions of the child’s reaction to the food with MNP; these document some children “liking” [19,20], “disliking” [20,21,22,23,24,25,26,27], or even refusing to consume [24,25,26,27,28,29] foods with added MNP. Few studies in programmatic settings confirm that MNPs do not affect organoleptic properties of food [30,31,32,33,34], and a greater number report changes to the color, taste or appearance of foods [19,26,27,35,36,37,38,39,40,41,42]. A handful of studies published in peer-reviewed journals report ratings for color, flavor, taste, and smell of foods with added MNP in community settings; some report perceptible changes with MNP with persistent high acceptability [43,44], whereas others report no changes [31,45].

Studies that report changes in the sensory properties of food with added MNP, including sourness, bitterness, acidity, and medicinal taste [36,38], attribute these changes either to improper preparation, such as use in addition to liquids such as soups, or beverages such as milk [21,25,37], or to poor quality of the MNP product [46]. The lipid-encapsulation coating, which prevents iron and other nutrients from dissolving into the food, can melt when added to hot foods, or float and stick to the cup when added to liquids [10,47]. Furthermore, MNP can be formulated with or without other vitamins and minerals, in addition to iron, vitamin A, and zinc, and multi-nutrient formulations, such as B-vitamins and copper [48], can impart adverse sensory properties. To our knowledge, there are no published MNP sensory evaluations in controlled settings in which the MNP are added to suitable foods (i.e., not hot or liquid foods) to confirm this assumption.

Several studies in programmatic settings in Indonesia have shown that *Taburia* is highly acceptable among most caregivers and children [49,50], and distribution of *Taburia* has shown to be effective in improving the nutritional status [50,51,52,53]. There are, however, reports of low adherence to the prescribed used of *Taburia* (21.9%) among children 6–24 months old from South Sulawesi Province [54], and a decrease in use from 100% in 2010 to 61.2% in 2011 among children from Palembang in the South Sumatra Province [55]. Furthermore, there are anecdotal reports of poor quality (bad smell upon opening sachet) and of the product altering the taste of foods of *Taburia* distributed in 2012 across 24 districts of Indonesia.

In this context, we sought to investigate the organoleptic properties of *Taburia* when added to foods commonly consumed by Indonesian children in a controlled study setting. The purpose of the sensory evaluation was to better understand the cause for reported child refusals of *Taburia*. We hypothesized that the addition of *Taburia* to foods commonly offered to Indonesian children 6–24 months of age does not affect the organoleptic properties of the foods, and does not reduce the acceptability of the foods, if prepared according to recommendations.

## 2. Materials and Methods

### 2.1. Study Design

We conducted sensory evaluations among caregivers and their infants and young children aged 6–24 months to determine the acceptability of *Taburia* and a comparison MNP. The sensory evaluations were conducted in two locations (Sidoarjo in East Java and Sukabumi in West Java) using two tasting panels, and included the following test meals:

Tasting panel 1: Caregivers and their infants and young children aged 6–24 months old

1. Plain rice porridge
2. *Taburia* mixed with rice porridge
3. Comparison MNP mixed with rice porridge

Tasting panel 2: Caregivers and their young children aged 13–24 months old

4. Foods commonly consumed by infants and young children
5. *Taburia* mixed with foods commonly consumed by infants and young children
6. Comparison MNP mixed with foods commonly consumed by infants and young children

The sensory evaluations were carried out consecutively with 10 min intervals, and were followed by face-to-face interviews with the caregivers in the local language (Indonesian or Javanese). Evaluations were conducted individually in a quiet area separated by cloth panels to ensure privacy. Participants were not given any food or drinks, other than water, for at least 60 min before the tasting sessions.

The study was double-blinded, since the formulation of the test meals was unknown to both the participants and interviewers. The study was approved by the University Atmajaya, Jakarta (registration number 312/III/LPPM-PM.10.05/03/2015). All study participants provided written consent.

### 2.2. Product Development

The comparison MNP product used was MixMe™, as formulated in 2010 for UNICEF and distributed through Save the Children and other agencies. The micronutrient premixes for both *Taburia* and the comparison MNP were supplied by DSM Nutritional Products Ltd. (Kaiseraugst, Switzerland). *Taburia* was produced in Singapore and packaged in Indonesia and the comparison MNP was produced and packaged in Malaysia.

The formulation of both products was very similar, but there were minor differences in composition, nutrient form (only for iodine), and levels of some nutrients (Table 1). The MNP products evaluated had 14 micronutrients in common. However, the comparison MNP contained copper, which is known to affect taste significantly, and *Taburia* contained pantothenic acid and vitamin K1, which are unlikely to affect taste [48]. The nutrient forms used were the same for all nutrients, except for iodine, which could affect saltiness or umami (potassium iodate for comparison MNP and potassium iodide for *Taburia*) [48]. There were negligible differences in the levels of vitamins A, E, and B12, niacin and selenium, whereas iodine content was higher in comparison MNP, and zinc content was slightly higher in *Taburia*, but this is unlikely to affect taste [48]. Other nutrients known to affect aroma or taste, such as B-vitamins, iron, and selenium, had the same nutrient level and form in both products. Both formulations contained a readily bioavailable form of encapsulated iron (ferrous fumarate). In addition, the *Taburia* blend contained tricalcium phosphate, which reduces sourness [48].

### 2.3. Study Location and Participants

The sensory evaluations were conducted in Sidoarjo (East Java) and Sukabumi (West Java) in April and May of 2015. Within each subdistrict, two villages were randomly selected for the study locations. Within each village, an integrated health post (Posyandu) was randomly selected for the recruitment of study participants. Each integrated health post (*n* = 4) provided a list of children in their registries, from which children aged 6–24 months were purposively selected to obtain equal sex and age group distributions (6–8 months, 9–11 months and 12–24 months).

To be eligible to participate, caregivers had to (i) be the primary caregiver of a child aged 6–24 months; (ii) be at least 18 years old; (iii) have completed junior high school; (iv) be living in the study area; and (v) have the capacity to understand the intent of the study, to make an informed decision regarding consent. Children had to be receiving semi-solid or solid complementary foods at least once per day for at least 30 days prior to the start of the study. Exclusion criteria for children were ailments that might affect appetite during the previous 7 days, dietary restrictions, or low weight-for-age (Z-score < −2 with respect to the WHO growth standards [56]) as reported by the integrated health post.

A total of 250 eligible child–caregiver pairs were invited to participate in the sensory evaluation. The response rate was 93%, and 232 child–caregiver pairs participated in the study (134 in Sidoarjo and 98 in Sukabumi).

### 2.4. Preparation of Test Meals

Child and caregiver acceptability of foods prepared with *Taburia* or comparison MNP was assessed using two distinct food vehicles commonly consumed by young children in the study area. Panel 1 participants were offered plain rice porridge, which was prepared at the test site using 200 g of rice per 1 L of water. Panel 2 participants were asked to prepare a meal commonly consumed by their child at their homes, and bring the meal to the test site. Local meals that were prepared included savory solid foods (*n* = 37) and semi-solid foods (*n* = 27), and a single child brought in rice porridge (*n* = 1). All savory meals included rice; other common ingredients included chicken, eggs, tofu, tempeh, and meatballs.

The test meals for the sensory evaluation were prepared from 50 g wet weight of prepared rice porridge or meal, and one 1 g sachet of MNP. The test meal was divided into two separate bowls, two-thirds was offered to the caregivers (~33 g) and one-third was offered to the child (~17 g). The MNP was mixed into the food at a temperature of 65 °C and served soon after. The ideal temperature of the food vehicle for the sensory evaluation was pre-tested by two researchers in a lab setting. *Taburia* was added to rice porridge samples at temperatures between 50 °C and 75 °C, at intervals of 2.5 °C, and the effect of temperature on the overall taste and appearance were evaluated. A temperature of 65 °C provided optimal conditions, at which *Taburia* dissolved well and there was no apparent negative effect on taste and coloring.

### 2.5. Caregiver Acceptability of Taburia or Comparison MNP Using Two Distinct Test Meals

**Figure 1:**
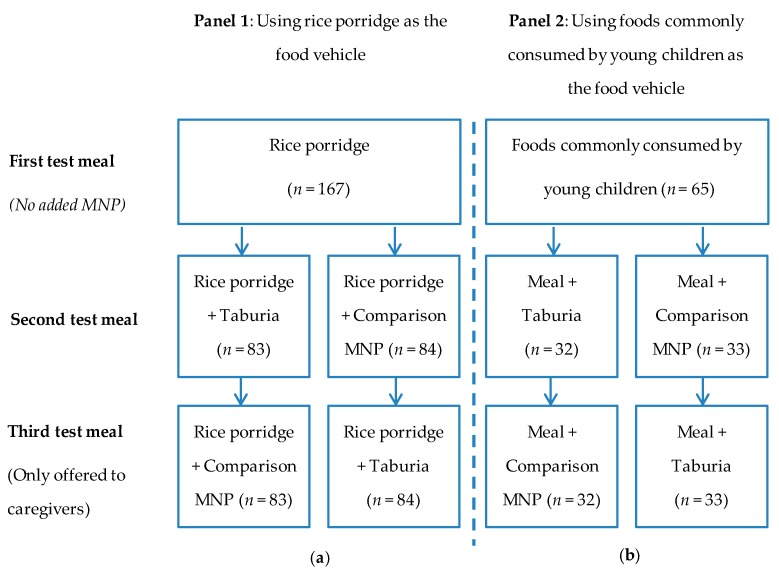
Feeding schedule for test meals offered to children and their caregivers. The offered meals were plain meal, meal with added *Taburia*, or meal with added comparison MNP. Children were offered two meals and caregivers were offered three meals. (**a**) Panel 1: Test meals among infants and young children aged 6–24 months and their caregivers (**b**) Panel 2: Test meals among infants and young children aged 13–24 months and their caregivers.

A sequential monadic test design was used, and participants were assigned to receive two different alternative orderings of treatments, as shown in Figure 1. Food samples were placed in uniform plastic cups and served with a separate plastic spoon to ensure no carryover of sensory properties. Participants were instructed to taste a single spoonful of each test meal, and were given a cup of water to rinse their mouth between each tasting. They were asked to evaluate the appearance (color), odor, texture, and taste (sweetness, saltiness, bitterness, and umami) of each sample on a nine-point hedonic scale for which 5 represented “*just about right*”. Overall appearance and taste were rated on a nine-point hedonic scale, for which 9 represented the most liked. In addition, participants were asked whether the sample had an aftertaste, and whether they liked the aftertaste.

### 2.6. Child Acceptability of Taburia or Comparison MNP Using Two Distinct Test Meals

Child acceptability of foods prepared with *Taburia* or comparison MNP was determined by direct observation by the caregiver. Rice porridge was used as the food vehicle for the sensory evaluation among infants and young children aged 6–24 months old in Sidoarjo (*n* = 134) and Sukabumi (*n* = 33). Meals commonly consumed by young children were used among children aged 13–24 months in Sukabumi (*n* = 65).

Children were offered a test meal (rice porridge or a meal commonly consumed by young children) followed by the same test meal with added MNP. The added MNP was *Taburia* for half the children and comparison MNP for the other half (Figure 1).

Children were offered a single spoonful of each test meal, then were continued to be fed by the caregiver. The caregiver and the interviewer observed the child’s reaction to the foods offered, and were asked to rate the child’s likability of the food using a 9-point hedonic scale (with 1 as ‘strongly dislike’ and 9 as ‘strongly like’) and record any further observations.

### 2.7. Infant and Young Child Feeding Practices

The sensory evaluation was followed by a face to face interview using a structured survey tool to determine infant and young child feeding practices, including current breastfeeding status, most commonly consumed complementary foods, and meal frequency.

### 2.8. Statistical Analysis

Statistical analyses were conducted using IBM SPSS Statistic Version 21.0 (IBM Corp., Armonk, NY, USA). Median caregiver ratings of key organoleptic properties of test meals and overall taste and appearance of the test meals (i.e., food vehicle, food vehicle + *Taburia*, or food vehicle + comparison MNP) were compared by non-parametric Friedman’s two-way analysis of variance by ranks. When significant differences were observed (*p* ≤ 0.05), Wilcoxon signed-rank tests on different combinations of the three test meals were carried out to determine the individual differences between them, and *p*-values were adjusted for multiple comparisons using Bonferroni adjustment.

Median scores for infant and young child overall liking of the plain meals versus meals with added *Taburia* and plain meals with added comparison MNP were compared using non-parametric Kruskal–Wallis test. Proportions of infants and young children who disliked, were neutral towards, or liked the test meals, and the proportion of children who rejected the test meals, were compared using a chi-squared test.

## 3. Results

### 3.1. Socio-Demographic Characteristics 

The socio-demographic characteristics of study participants are shown in Table 2. All caregivers were the mother of the index child. Panel 1 participants (*n* = 167) were from Sidoarjo (80%) and Sukabumi (20%), and infants were aged between 6–24 months, with equal representation among the three age groups and sexes. Panel 2 participants (*n* = 65) were from Sukabumi, and children were aged 13–24 months. Mean ± SD caregiver age was 29.7 ± 5.7 years for panel 1 caregivers, and 28.5 ± 6.5 years for panel 2 caregivers, with a greater proportion of younger women (aged < 25 years) among panel 2 participants. Although formal education levels differed between panels, with a higher proportion of caregivers who completed junior high school only among panel 2 participants, self-reported monthly incomes did not differ.

### 3.2. Caregiver Acceptability of Taburia or Standard MNP in Two Distinct Test Meals

**Figure 2:**
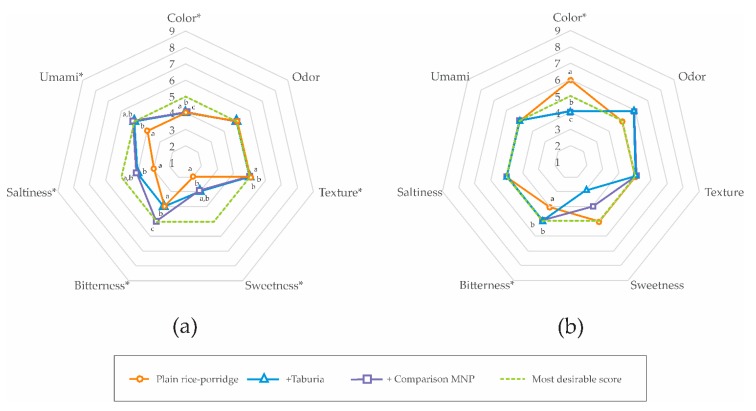
Caregiver evaluation of key organoleptic properties of test meals on a 9-point scale for which 5 represents “*just about right*”. (**a**) Panel 1: using rice porridge as the food vehicle, *n* = 167; (**b**) Panel 2: using foods commonly consumed by infants and young children as the food vehicle, *n* = 65. * Significant difference between test meals (i.e., food vehicle, food vehicle + *Taburia*, food vehicle + comparison) using non-parametric Friedman’s two-way analysis of variance by ranks (*p* ≤ 0.05). Different superscript letters indicate significant differences between test meals using Wilcoxon signed-rank tests.

Caregiver evaluation of key attributes of test meals using a 9-point scale showed that most sensory attributes that may affect the acceptability of meals changed significantly with the addition of MNP (Table 3 & Figure 2). Using rice porridge as a food vehicle (panel 1), caregivers were able to detect differences between test meals for color, texture, sweetness, bitterness, saltiness, and umami (*p* < 0.05). With the exception of color and bitterness, ratings were more desirable (i.e., closer to a “*just about right*” rating) for rice porridge with added *Taburia*. Comparison MNP affected the score for color and bitterness more than *Taburia*. Using common meals as a food vehicle (panel 2), caregivers were able to detect differences between test meals for color and bitterness. All ratings with significant differences were less desirable for meals with added MNP.

**Figure 3:**
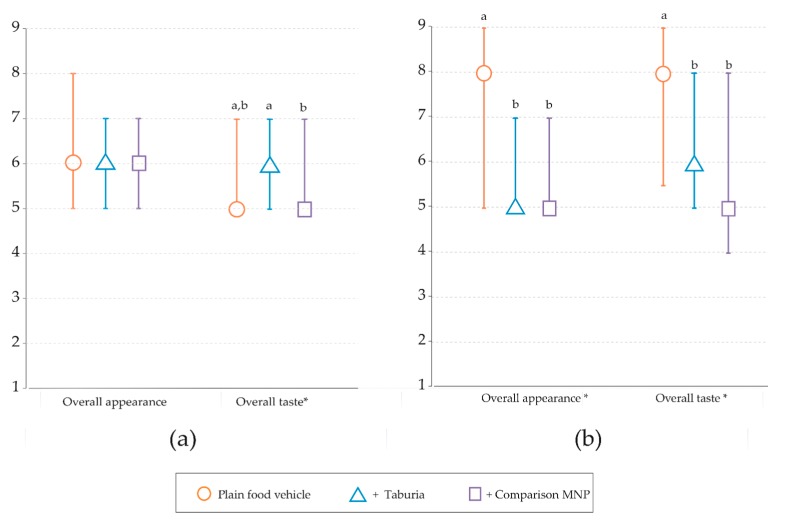
Caregiver evaluation of overall taste and overall appearance of test meals on a 9-point scale, for which 9 represents the most liked. (**a**) Panel 1: using rice porridge as the food vehicle, *n* = 167; (**b**) Panel 2: using foods commonly consumed by infants and young children as the food vehicle, *n* = 65. * Significant difference between test meals (i.e., food vehicle, food vehicle + *Taburia*, food vehicle + comparison MNP) using non-parametric Friedman’s two-way analysis of variance by ranks (*p* ≤ 0.05). Different superscript letters indicate significant differences between test meals using Wilcoxon signed-rank tests.

Using rice porridge as a food vehicle (panel 1), no significant differences were observed between the overall appearance of the test meals (*p* = 0.018), but ratings for overall taste were significantly higher for rice porridge with added *Taburia* when compared to both the plain rice porridge and rice porridge with added comparison MNP (Table 3 & Figure 3). Using common meals as a food vehicle (panel 2), the overall rating of both overall appearance and taste were higher for the plain meals, and no differences were observed between *Taburia* and comparison MNP.

Approximately one-third of caregivers (36%) reported an aftertaste for plain rice porridge, and approximately 40% reported an aftertaste for rice porridge with added MNP (42% for *Taburia* and 40% for comparison MNP) (*p* = 0.516). Dislike of the aftertaste was greater for rice porridge with added MNP when compared to plain rice porridge. The proportion of caregivers who reported disliking the aftertaste was 24% for plain rice porridge, 36% with added *Taburia*, and 60% with added comparison MNP (*p* = 0.001). Just over half the caregivers (55%) reported an aftertaste after consuming meals commonly consumed by young children, significantly more reported an aftertaste with one or the other added MNP (65% for *Taburia* and 63% for comparison MNP) (*p* = 0.513). Dislike of the aftertaste was lower for meals with added MNP. The proportion of caregivers who reported disliking the aftertaste was 42% for plain meals, 39% with added *Taburia*, and 30% with comparison MNP (*p* = 0.260).

None of the test meals were rejected by the caregivers. A comparison of preference of test meals shows that more caregivers preferred rice porridge with added *Taburia* (25%) over plain rice porridge (16%) or rice porridge with added comparison MNP (15%), and a further 9% reported no preference between rice porridge meals (*p* = 0.058). The remaining 36% of caregivers rated at least two meals equally. When using common child meals as the food vehicle, more caregivers preferred plain meals (43%) over meals with added *Taburia* (22%), and even fewer preferred meals with added comparison MNP (8%). A further 9% reported no preference between meals and the remaining 19% rated at least two meals equally (*p* < 0.001).

### 3.3. Child Acceptability of Taburia or Comparison MNP in Two Distinct Test Meals

The median child liking score (using 9-point scale, and 9 being the most liked) based on the caregiver’s observation was 7 (interquartile range (IQR) 5–9) for plain rice porridge, and lower for rice porridge with added *Taburia* 6 (IQR 5–8), and with added comparison MNP 5.5 (IQR 4–8) (*p* = 0.085). The median child liking score for commonly consumed meals was 6 (IQR 8–9) for plain meals, and higher for meals with added *Taburia* 7 (IQR 5–9), and with added comparison MNP 7 (IQR 5–8) (*p* = 0.211). A similar pattern was observed based on interviewers’ observations (data not shown).

When the child liking scores were categorized as dislike (score 1 to 4), neutral (score 5), and like (score 6 to 9), differences were observed between meals (Table 4). Among children in panel 1, fewer disliked, and more liked the plain rice porridge, compared to rice porridge with added *Taburia* (*p* = 0.001) or added comparison MNP (*p* < 0.001). Similar trends were observed among children in panel 2.

Reported reasons for low scores (score 1 to 4) included the following observations: child spat out the food, was restless, did not eat enthusiastically, ate small amounts of food or had an expression indicating dislike of the food. Reported reasons for high scores (score 6 to 9) included the following observations: child ate enthusiastically, looked happy, asked for more food, is calm and seems to like the food.

A significant proportion of children rejected the test meals (i.e., spat out the food) and the proportion of children who rejected the meals differed significantly by test meal. Among children in panel 1, the rejection rate was 19% for plain rice porridge, 13% for rice porridge with added *Taburia*, and 16% for rice porridge with added comparison MNP (*p* = 0.255). Among children in panel 2, the rejection rate was 11% for plain meals, 5% for meals with added *Taburia*, and 14% for meals with added comparison MNP (*p* = 0.195).

A comparison of child preference of rice porridge test meals as reported by caregivers shows that one-quarter of children did not show a preference between rice porridge test meals, and equal proportions seemed to prefer the rice porridge test meals with added MNP (either *Taburia* or comparison MNP). Of the 83 children who tasted both plain rice porridge and rice porridge with added *Taburia*, 39% seemed to prefer plain rice porridge, 36% seemed to prefer rice porridge with added *Taburia*, and 25% seemed not to have a preference (*p* < 0.001). Of the 84 children who tasted both plain rice porridge and rice porridge with added comparison MNP, 36% seemed to prefer plain rice porridge, 36% seemed to prefer rice porridge with added comparison MNP and 28% seemed not to have a preference (*p* < 0.001).

A comparison of child preference of common food test meals, as reported by caregivers, shows distinct differences between MNP products. Of the 32 children who tasted plain meals and meals with added *Taburia*, 52% seemed to prefer plain meals, 30% seemed to prefer meals with added *Taburia* and 18% seemed not to have a preference (*p* = 0.010). Of the 33 children who tasted plain meals and meals with added comparison MNP, 28% seemed to prefer plain meals, 38% seemed to prefer rice porridge with added comparison MNP, and 34% seemed not to have a preference (*p* = 0.005).

There were no reports among caregivers of unwillingness to offer *Taburia* or comparison MNP to their child as a consequence of disliking the sensory attributes of the supplements.

### 3.4. Infant and Young Child Feeding Practices

The proportion of children who were breastfeeding was 74% at 6–9 months, 63% at 10–12 months, and 55% at 13–24 months of age in Sidoarjo; and 77% at 6–9 months, 87% at 10–12 months, and 69% at 13–24 months of age in Sukabumi. At 6–9 months, infants were typically offered rice porridge, soft fruits, biscuits, and crackers. At 10–12 months caregivers started to combine rice porridge with steamed rice and side dishes, such as meats. Infants were also offered fruit juices and sliced fruits, biscuits, bread, and other snacks. At 13–24 months, young children were offered a variety of foods, and the diet was very similar to the diet of the family.

The predominant food for infants aged 6–9 months was rice porridge, with 82% of caregivers reporting that they offered rice porridge daily. The proportion of children consuming rice porridge daily decreased with age, with half of the 10–12 month olds and one-third of the 13–24 month olds consuming rice porridge daily. The predominant foods for children aged 10 months and older were rice and fruits.

In Sidoarjo, caregivers often worked and children were commonly offered instant foods and fed by their grandmothers, whereas caregivers in Sukabumi commonly fed their child home-made food themselves. Meal frequency and serving size increased with age from two meals a day and 3–8 spoons per meal at 6–9 months, to three meals a day and 10–12 spoons per meal at 10–24 months.

## 4. Discussion

This sensory evaluation confirms documented reports of changes to the organoleptic properties of foods with the addition of *Taburia*, even when following preparation instructions (i.e., not added to hot food or liquids). Most sensory attributes that may affect the acceptability of the test meals changed significantly, and changes differed according to the food vehicle used. *Taburia* was found to negatively affect the color and bitterness of foods, but also enhance texture, sweetness, saltiness, umami taste, and overall taste of plain rice porridge, resulting in the preference of rice porridge with added *Taburia* over plain rice porridge. *Taburia* also affected the organoleptic properties of meals commonly consumed by young children, but ratings were less desirable for meals with added *Taburia*. Scores for organoleptic properties of meals with added comparison MNP were not significantly different from plain meals, with the exception of color and bitterness, which affected the overall ratings of appearance and taste negatively. Regardless of perceived changes, caregivers in our study did not reject either MNP, and the acceptability of *Taburia* was found to be high. Our findings confirm previous reports that perceived changes in organoleptic properties generally did not deter caregivers from offering MNP to their children [18,35,38,44]. Although, a few studies report that dislike of MNP discouraged use, and was reported as the most common reason for not giving all of the MNP sachets to the child [22,25].

Most studies reporting an effect of MNP on taste describe unpleasant attributes, such as sourness, bitterness, acidity, or medicinal taste [36,38], but a few describe changes in taste that are desirable. Jefferds et al. [19] report children liking the taste of MNP (Sprinkles) eliciting children to request MNP to be added to their meals. Olney et al. [57] report that caregivers liked the taste and smell of MNP, or that the MNP had no taste or smell. Our study shows that *Taburia* can actually enhance the taste of plain rice porridge. In our sensory evaluation, most changes to the taste of foods with the addition of MNP were perceived as being positive, whilst others, such as bitterness, were off-putting.

The perceived differences in sensory attributes of *Taburia* and comparison MNP, both supplied by DSM Nutritional Products Ltd. (Kaiseraugst, Switzerland), can be attributed to slight differences in composition. The comparison MNP contained copper, which is known to affect taste significantly, and different forms of iodine were used (Table 1) [48]. In addition, the *Taburia* blend contains tricalcium phosphate, which reduces sourness. Our sensory evaluation of *Taburia* and comparison MNP illustrates the consequence of nutrient composition, dose, and form, on sensory attributes that could affect acceptability of the product. Determining the most effective and cost-effective dose for anemia reduction and other outcomes, and the selection of the best combination/number of nutrients included, was identified as a research gap by WHO [9]. In addition to the best composition of MNP for biological impact, further research on the best composition to improve organoleptic properties of food mixed with MNP could improve acceptability and adherence outcomes [46].

Our findings must be interpreted within the limitations of the study. The Likert scale was not tested beyond our study respondents, and as such, the scoring proportions (i.e., percentage of respondents providing a particular rating) may not be valid for the broader Indonesian population. However, use of the same Likert scale, researchers, and respondents, increases reliability of the measurements and estimations of rating differences across test meals. Further, distributions of rating responses were normal and narrow, which indicates that the Likert scale was reliable, and reflects true differences among the test meals. Acceptability among infants and young children is difficult to evaluate, and is subject to caregivers’ and researchers’ interpretations. In our study, plain rice porridge was used as a food vehicle, but children in Indonesia typically consume rice porridge accompanied by other foods, such as chicken and vegetables. It is possible that rice porridge prepared with MNP as well as additional ingredients may have higher acceptability [18]. The findings of our study may not apply to other MNP products with varying formulations, and in a different context, where other foods are commonly consumed by infants and young children.

In our study, ratings of organoleptic properties varied depending on the food vehicle used. Rice porridge was shown to be a better food vehicle than the meals provided by the caregivers when asked to bring meals commonly consumed by their children. The meals provided were often solid or liquid, which makes it difficult to add MNP. Furthermore, mixed, home-cooked meals are more likely to have ingredients or nutrients that may interact with MNP than plain rice porridge. This highlights the importance of promoting the use of foods into which MNP can be added easily. At breakfast, rice porridge is commonly consumed among younger infants, whereas rice is more commonly consumed at a later stage. Other foods that are commonly consumed and are appropriate for adding MNP include fruits and vegetables. Although our evaluation demonstrated that rice porridge is a good vehicle for MNP, the effect of adding MNP to rice porridge served at temperatures above 65 °C warrants further investigation, as the lipid coating around the nutrient will melt and cause changes to the color of the food and affect taste. In Indonesia, caregivers ensure that foods served to young children are not too hot by tasting or touching it before offering.

The fact that *Taburia* can be detected when added to foods commonly consumed by children, and affects sensory attributes of the foods, while at the same time improving the nutrient adequacy of the meal, should be well understood by caregivers. *Taburia* was shown to enhance the taste of plain rice porridge, which can help the promotion of the product. Informing the caregivers that slight changes in color are normal can be reassuring. In addition, choice of food vehicle and proper preparation of MNP are critical to enhancing the acceptability, and should be key factors conveyed to caregivers during interventions. Very specific instructions—“do not use in liquids or hot food”—need to be given very explicitly. Following these instructions will prevent changes to color and taste, and prevent the powder clumping or not dissolving. Correct food preparation with MNP, varying the foods fed to children mixed with MNP, and encouraging caregivers not to assume the child will dislike food mixed with MNP, are strategies that are likely to help reduce the perception of child refusals. Furthermore, cultural practices, socio-economic factors, the availability of food, and infant and young child feeding practices, also impact the acceptability of *Taburia* and adherence to prescribed use.

Few studies report ratings for color, flavor, taste, and smell of foods with added MNP in community settings [31,43,44,45], and to the best of our knowledge, this is the first publication in a peer-reviewed journal reporting a sensory evaluation of the organoleptic properties of food with added MNP in a controlled setting. Our findings provide information that can help with developing strategies to improve acceptability of *Taburia* in Indonesia, but also have implications beyond the Indonesian context. We demonstrated that MNP can affect organoleptic properties of foods, even when prepared according to guidelines. This needs to be acknowledged and taken into consideration for product development and communication strategies promoting adherence.

## 5. Conclusions

We conclude that although organoleptic changes to foods are perceived with the addition of *Taburia*, these changes are unlikely to be the cause of reported adherence problems in Indonesia. Based on global research and program experience, it is more likely that caregivers were not adequately instructed regarding the preparation of *Taburia*, and mixing it with inappropriate foods (or liquids) or at too high a temperature [18].

## Figures and Tables

**Table 1:** Nutrient composition of comparison MNP and *Taburia* in relation to nutrient requirements, and expected effect of nutrient on taste and color.

Nutrient	Nutrient Form		Nutrient Composition per 1 g MNP Sachet		Daily Requirements WHO RNI ^4^		Nutrient Affects Taste	Nutrient Affects Color
	Comparison MNP ^1^	Taburia ^2,3^	Comparison MNP ^1^	Taburia ^2^	7–12 months	1–3 years		
Vitamin A	Acetate		400 µg RE	417 µg RE	400 µg RE	400 µg RE		Hardly visible colored particles ^5^
Vitamin D3	Cholecalciferol		5 µg	5 µg	5 µg	5 µg		
Vitamin E	dl-alpha-Tocopheryl acetate		5 mg	6 mg	2.7 mg α-TE	5.0 mg α-TE		
Folic acid	Folic acid		0.15 mg	0.15 mg	80 µg DFE	150 µg DFE		Hardly visible colored particles ^5^
Niacin	Niacinamide		6 mg	5 mg	–	–		
Pantothenic acid	–	D-Calcium Pantothenate	–	3 mg	–	–		
Vitamin B1	Thiamine Mononitrate		0.5 mg	0.5 mg	0.3 mg	0.5 mg	A slightly “soupy” or “yeasty” taste ^5^, known to affect aroma or taste ^6^	
Vitamin B12	Cyanocobalamin		0.9 µg	1 µg	0.7 µg	0.9 µg		Hardly visible colored particles ^5^
Vitamin B2	Riboflavin		0.5 mg	0.5 mg	0.4 mg	0.5 mg	Slightly bitter for bitter-sensitive persons ^5^, known to affect aroma or taste ^6^	May add a shade of yellow ^5^
Vitamin B6	Pyridoxine		0.5 mg	0.5 mg	0.3 mg	0.5 mg		
Vitamin C	Ascorbic Acid		30 mg	30 mg	30 mg	30 mg	Slightly acid ^5^	
Vitamin K1	–	Phytonadione	–	20 µg	5 µg	10 µg		
Copper	Copper Sulfate, anhydrous	–	0.56 mg	–	–		Metallic taste ^5^, known to affect taste significantly ^6^	Hardly visible colored particles ^5^
Iodine	Potassium Iodate	Potassium Iodide	90 µg	50 µg	90 µg	90 µg	Could affect saltiness or umami ^6^	
Iron	Ferrous Fumarate		10 mg	10 mg	9.3 mg ^7^	5.8 mg ^7^	Metallic taste ^5^, known to affect aroma or taste ^6^	Fe-Fumarate colors in the form of violet ^5^ spots
Selenium	Sodium Selenite		17 µg	20 µg	10 µg	17 µg	Known to affect aroma or taste ^6^	
Zinc	Zinc Gluconate		4.1 mg	5 mg	4.1 mg ^8^	4.1 mg ^8^	Metallic taste ^5^	

α-TE, α-tocopherol equivalents; DFE, dietary folate equivalent; MNP, micronutrient powder; RE, retinol equivalent; RNI, recommended nutrient intakes. ^1^ Formulated in 2010 for UNICEF, premix supplied by DSM Nutritional Products Ltd. (formula FT101644AP); ^2^ Developed in 2006 by the National Institute for Health Research and Development (NIHRD), Indonesia, premix supplied by DSM Nutritional Products Ltd. (formula FT071902AP); ^3^ In addition, the *Taburia* blend contains tricalcium phosphate, which reduces sourness; ^4^ WHO and FAO 2004 [14]; ^5^ Taken from the HF-TAG Quality Manual on Micronutrient Powders [47]; ^6^ [48]; ^7^ Assuming 10% bioavailability; ^8^ Assuming moderate bioavailability. “–“ refers to none.

**Table 2:** Socio-demographic characteristics of study participants.

Socio-Demographic Characteristics	Panel 1: Using Rice Porridge as the Food Vehicle		Panel 2: Using Foods Commonly Consumed by Young Children as the Food Vehicle	
	(*n* = 167)		(*n* = 65)	
**Infant Characteristics**				
**District**				
Sidoarjo	134	80%	–	–
Sukabumi	33	20%	65	100%
**Sex**				
Boy	81	49%	33	51%
Girl	86	51%	32	49%
**Age in months**				
6–9 months	55	33%	–	–
10–12 months	54	32%	–	–
13–24 months	58	35%	65	100%
**Caregiver Characteristics**				
**Age in years**				
18–25 years	43	26%	30	46%
26–30 years	56	34%	14	22%
31–35 years	39	23%	11	17%
36+ years	29	17%	10	15%
**Formal education**				
Junior high school	74	44%	53	82%
Senior high school or equivalent	82	49%	10	15%
Further education	11	7%	1	2%
**Monthly household income**				
High (more than 187 USD/month)	34	20%	15	23%
Medium (112 to 187 USD/month)	66	40%	26	40%
Low (less than 112 USD/month)	67	40%	24	37%

“–“ Refers to none.

**Table 3:** Caregiver ratings of key organoleptic properties of test meals (i.e., food vehicle, food vehicle + *Taburia*, food vehicle + comparison MNP) ^1^.

Organoleptic Properties	Plain Meal		+*Taburia*		+Comparison MNP		*p*-Value ^2^
	Median	(IQR)	Median	(IQR)	Median	(IQR)	
**Panel 1:** Using rice porridge as the food vehicle (*n* = 167)							
Color	4 ^a^	(4–7)	4 ^b^	(3–5)	4 ^c^	(2–4)	<0.001
Odor	5	(5–5)	5	(5–6)	5	(5–6)	0.156
Texture	5 ^a^	(4–5)	5 ^b^	(5–7)	5 ^b^	(5–7)	<0.001
Sweetness	2 ^a^	(1–5)	3 ^b^	(1–5)	3 ^a,b^	(1–5)	0.001
Bitterness	4 ^a^	(4–7)	4 ^b^	(4–7)	5 ^c^	(4–8)	<0.001
Saltiness	3 ^a^	(2–5)	4 ^b^	(2–5)	4 ^a,b^	(2–5)	0.005
Umami	4 ^a^	(2–5)	5 ^b^	(4–5)	5 ^a,b^	(3–5)	0.002
Overall appearance	6	(5–8)	6	(5–7)	6	(5–7)	0.118
Overall taste	5 ^a,b^	(5–7)	6 ^a^	(5–7)	5 ^b^	(5–7)	0.012
**Panel 2:** Using foods commonly consumed by young children as the food vehicle (*n* = 65)							
Color	6 ^a^	(4–7)	4 ^b^	(3.5–5)	4 ^c^	(2–4)	<0.001
Odor	5	(5–8)	6	(5–8)	6	(5–8)	0.607
Texture	5	(3–7)	5	(3–6.5)	5	(3–6)	0.600
Sweetness	5	(3–5)	3	(2–5)	4	(1.5–5.5)	0.075
Bitterness	4 ^a^	(4–5)	5 ^b^	(4–7)	5 ^b^	(4–8)	0.003
Saltiness	5	(3–5)	5	(3–5)	5	(4–5)	0.316
Umami	5	(5–7)	5	(5–7)	5	(4–7)	0.193
Overall appearance	8 ^a^	(5–9)	5 ^b^	(5–7)	5 ^b^	(5–7)	<0.001
Overall taste	8 ^a^	(5.5–9)	6 ^b^	(5–8)	5 ^b^	(4–8)	<0.001

IQR: interquartile range; MNP, micronutrient powder. ^1^ 5 on a 9-point scale represents “*just about right*”; ^2^ *p*-value for differences between three test meals (i.e., food vehicle, food vehicle + *Taburia*, food vehicle + standard MNP) using non-parametric Friedman test for repeated measures (*p*-value < 0.05); Within a row, values with different superscript letters are significantly different using Wilcoxon signed-rank test for pair-wise comparisons (Bonferroni adjusted) (*p*-value < 0.05).

**Table 4:** Overall child liking test meals (i.e., food vehicle, food vehicle + *Taburia*, food vehicle + comparison MNP).

Child Liking Score Using 9-Point Scale ^1^	Proportion (%)			*p*-Value ^2^	
**Panel 1:** Using rice porridge as the food vehicle (*n* = 167) ^3^	Rice porridge (*n* = 167)	Rice porridge + *Taburia* (*n* = 83)	Rice porridge + Comparison MNP (*n* = 84)	Rice porridge vs +*Taburia*	Rice porridge vs + Comparison MNP
Caregiver’s observation					
Dislike (score 1–4)	14%	20%	29%	0.001	<0.001
Neutral (score 5)	25%	24%	21%		
Like (score 6–9)	61%	55%	50%		
Interviewer’s observation					
Dislike (score 1–4)	17%	28%	29%	<0.001	<0.001
Neutral (score 5)	23%	19%	18%		
Like (score 6–9)	59%	53%	54%		
**Panel 2:** Using foods commonly consumed by infants and young children as the food vehicle (*n* = 65) ^4^	Common meal (*n* = 65)	Meal + *Taburia* (*n* = 33)	Meal + Comparison MNP (*n* = 32)		
Caregiver’s observation					
Dislike (score 1–4)	8%	6%	15%	0.001	0.116
Neutral (score 5)	14%	22%	15%		
Like (score 6–9)	78%	72%	70%		
Interviewer’s observation					
Dislike (score 1–4)	8%	13%	18%	0.098	0.019
Neutral (score 5)	8%	19%	15%		
Like (score 6–9)	85%	69%	67%		

^1^ A score 9 represents the most liked; ^2^ *p*-value for difference between test meals (i.e., food vehicle, food vehicle + *Taburia*, food vehicle + comparison MNP) using chi-square test; ^3^ Among infants and young children aged 6–24 months old; ^4^ Among young children aged 13–24 months old.
